# Reply: Flawed methods explain the effect of mammography screening in Nijmegen

**DOI:** 10.1038/bjc.2011.265

**Published:** 2011-07-26

**Authors:** A L M Verbeek, M J M Broeders, G van Schoor, S M Moss, J D M Otten, R Donders, E Paap, G J den Heeten, R Holland

**Affiliations:** 1 Department of Epidemiology, Biostatistics and HTA, Radboud University Nijmegen Medical Centre, PO Box 9101, Nijmegen 6500 HB, The Netherlands; 2 Cancer Screening Evaluation Unit, Institute of Cancer Research, 15 Cotswold Road, Sutton, Surrey 2M2 5NG, UK; 3 National Expert and Training Centre for Breast Cancer Screening, PO Box 6873, Nijmegen 6503 GJ, The Netherlands

**Sir**,

We thank Dr Jørgensen for his interest in our study and welcome the opportunity to address the points of concern that he has raised.

We did not use the results of our case-referent design to ‘claim’ an effect of screening; rather we interpreted the study results to be consistent with a substantial effect of mammographic screening on breast cancer mortality. We believe that the case-referent approach offers a valid and efficient methodological framework for estimating the impact of service screening programmes in the routine healthcare environment (Verbeek and Broeders, 2010). The design of a case-referent study should benefit from state-of-the-art methodological insights (Paap *et al*, 2011). In fact, earlier studies corroborate that well-designed observational studies produce results that are similar to those from randomised controlled trials and do not yield a systematic bias in the effect estimates (Demissie *et al*, 1998; Concato *et al*, 2000). It is clear from the current scientific debate that some researchers are still not convinced that observational studies can be considered reliable designs for evaluating the impact of screening because of their vulnerability to bias (Gøtzsche and Jørgensen, 2005; Corder, 2010).

A case-referent study is a research design with an efficiency gain that comes from taking a sample of the denominator experience, that is, the population is invited to be screened over time, rather than having to observe the entire denominator experience (Rothman, 2001). The growing understanding of these studies explain the methodological differences between the early 1984 (Verbeek *et al*, 1984) and the 2011 design (van Schoor *et al*, 2011). The so-called discrepancy with the 28% mortality reduction estimated for the 1975–1991 observation period is largely explained by the difference in follow-up time. The early study included only deaths and screening in the period 1975–1981, whereas our later analysis includes deaths up to 1991 (as well as screening in this later period). The numbers in the earlier analysis are also comparatively small. Nevertheless, when limiting van Schoor's analysis to the period 1975–1981, included in the earlier 1984 analysis, the 2011 design yields an effect estimate of 69% (OR=0.31, 95% CI: 0.12–0.79) as shown in Table 1. Age-specific estimates from the 2011 study are further quite comparable with those estimated from the second case–control study published in 1985 (Verbeek *et al*, 1985).

Explanations as to why these large effects found in women who attend screening in Nijmegen do not translate into a breast-cancer mortality reduction at the population level have been reported previously (Broeders *et al*, 2001). The major constraint is the small size of the Nijmegen population (∼150 000 inhabitants), which results in considerable fluctuations in the annual numbers of breast cancer deaths over time. In addition, uptake of screening varies across individuals and migration in and out of Nijmegen cannot be accounted for in aggregated data. Both factors dilute the effect of screening at the population level.

The Malmö study used as an example to illustrate the ‘flawed results’ in the case–control design was actually used as illustration to the contrary in a study by Duffy *et al* (2002). This study showed that, after correction for non-compliance and selection bias, the intention to treat (ITT) effect was estimated as 1.03 (0.59–1.79), close to the observed RR of 0.96 (0.68–1.35) from the RCT. Other examples from this study show similar consistencies for case–control and RCT estimates. The same is true for the case–control evaluation of the UK Trial of Early Detection of Breast Cancer, which demonstrated that a case–control study of invited *vs* non-invited women produced similar results to those of the trial as a whole; again selection bias accounts for differences in the attender *vs* non-attender comparison (Moss *et al*, 1992). Both studies thus elegantly demonstrate that case–control studies do produce equivalent results – as also concluded by Demissie *et al* (1998) – once appropriate adjustments have been made.

In general, self-selection is the most difficult form of bias to deal with in the context of case-referent studies on cancer screening. Because participation in service screening is voluntary, selection factors related to both the likelihood of being screened and the risk of dying from cancer may confound the estimates of efficacy. Reviews of the literature show that the presence and direction of the bias vary from study to study (Moss, 1991; Cronin *et al*, 1998). Some studies report a higher mortality of breast cancer among non-attenders, thus overestimating the protective effect of screening; others find non-attenders to be at lower risk of breast cancer death, which would lead to underestimation of the effect of screening. In The Netherlands, we found a lower baseline risk in women who do not attend screening in the regions close to Nijmegen. Adjusting our odds ratio for self-selection bias using this correction factor of 0.84 (95% CI: 0.58–1.21), would thus result in an even larger effect (Paap *et al*, 2010).

It is interesting to note that the quote put forward by Dr Jørgensen from the IARC monograph refers to all observational studies, not just case–control studies. In contrast to this quote, Jørgensen places his trust in trend studies, as well as incidence-based mortality studies, both observational designs by nature. In addition, it is curious that studies using a design very similar to Kalager *et al* (2010) study and showing an impressive breast-cancer mortality reduction in relation to mammographic screening are not discussed (Olsen *et al*, 2005; Hellquist *et al*, 2011).

With his letter to the editor, colleague Jørgensen has prompted us to review our studies and the interpretation of their findings. We continue to believe that case-referent studies are and will remain a valid tool for the evaluation of cancer service screening.

## Figures and Tables

**Table 1:** The odds ratio of breast cancer death for screened *vs* unscreened women invited in Nijmegen in the period 1975–1982

**Calendar period^a^**	**Study**	**Study**
1975–81	Verbeek *et al* (1984)	Van Schoor *et al* (2011) b
		
*Age group*		
35+	0.48 (0.23–1.00)	
50–69		0.31 (0.12–0.79)
1975–82	Verbeek *et al* (1985)	Van Schoor *et al* (2011) b
		
*Age group*		
35–49	1.23 (0.31–4.81)	
50–64	0.26 (0.10–0.67)	0.32 (0.14–0.80)
65–69		0.75 (0.09–6.47)
65+	0.81 (0.23–2.75)	
		
Overall	0.51 (0.26–0.99)	0.37 (0.12–0.79)

a The calendar period comprises both the years of screening invitation and the time period of case ascertainment.

b These are the results of an extended analysis adapted to Verbeek's study periods.
